# Adjuvants Alter the Setting Behavior of a Ceramic Bone Graft Substitute: Implications for the Laboratory and Operating Room

**DOI:** 10.3390/ma19091873

**Published:** 2026-05-01

**Authors:** Felix Lamadé-Dootz, Nick Mattern, Sanja Kalmus, Alma Aubert, Paul Alfred Grützner, Jonas Armbruster, Holger Freischmidt

**Affiliations:** BG Klinik Ludwigshafen, Department for Orthopaedics and Trauma Surgery, Heidelberg University, Ludwig-Guttmann-Str. 13, 67071 Ludwigshafen, Germany; felix.dootz@bgu-ludwigshafen.de (F.L.-D.); nick.mattern@bgu-ludwigshafen.de (N.M.); sanja.kalmus@bgu-ludwigshafen.de (S.K.); alma.aubert@bgu-ludwigshafen.de (A.A.);

**Keywords:** non-unions, bone defect, HACaS, artificial bone substitute, macro-indentation, Vicat test, Gillmore test, liquid-to-powder ratio, zoledronic acid, BMP-2

## Abstract

**Highlights:**

**What are the main findings?**

- Delayed adjuvant addition (2 min) accelerates setting behavior.
- The addition of zoledronic acid delays early setting of HACaS.
- Initial discrepancies in setting behavior diminish after 24 h in blood.

**What are the implications of the main findings?**

- Delayed addition within the tested protocol was associated with faster early hardening.
- Citrate-containing zoledronic acid solutions were associated with delayed setting in this assay.
- Early setting differences diminished after 24 h of blood incubation, but ZA (0 min) remained inferior.

**Abstract:**

Hydroxyapatite–calcium sulfate (HACaS) bone cements have been clinically established. Combining HACaS with an antiresorptive (zoledronic acid, ZA) and osteoanabolic agent (bone morphogenic protein 2; BMP-2) may enhance the performance of HACaS bone cements in challenging indications, but it must be ensured that this does not impair their setting and mechanical properties. This study established a Vicat/Gillmore-inspired indentation protocol to quantify force-based endpoints and the setting of HACaS with biological adjuvants. HACaS was mixed with or without ZA and/or BMP-2 at 0 min and after a 2 min pre-setting phase with reduced NaCl content (lower liquid-to-powder ratio). For each time point (3–90 min), three cylindrical pellets (Ø 4 mm, height 6 mm) underwent single indentation. Setting was defined as the maximum force at needle penetration, and endpoint hardness was defined as peak force at failure. For 24 h endpoints, specimens were incubated in blood at 37 °C. One-way ANOVA with Tukey’s H post hoc test was performed per time point (*n* = 3; 24 h endpoints *n* = 5). All 2 min protocols showed accelerated setting, consistent with the initial lower liquid-to-powder ratio. ZA significantly delayed setting and remained lowest at 90 min and after 24 h in blood. Mixing sequence and vehicle composition critically influenced early mechanical properties and should be considered in the further preclinical evaluation of HACaS with osteoanabolic or antiresorptive agents.

## 1. Introduction

Artificial bone replacement materials are indispensable in modern fracture and reconstructive surgery. Bone substitutes are the second most frequently transplanted tissues worldwide [1]. An ideal bone substitute combines osteoconduction, osteoinduction, and, given the frequent occurrence of infections in bone-healing disorders, a bactericidal component [2]. Osteoconduction describes the migration and activity of osteoblasts and osteoclasts along a material, resulting in the formation of new bone, whereas osteoinduction describes the ability to stimulate cells to form bone [3].

Calcium sulfate/calcium phosphate composites have been clinically established [4]. Hydroxyapatite-based materials are widely regarded as promising biomaterials in various medical fields, including bone tissue engineering and interdisciplinary regenerative applications [5,6]. A hydroxyapatite–calcium sulfate (HACaS) substitute has demonstrated high rates of bony bridging in small defects and has also been used in previous animal experiments; therefore, reliable preliminary experience exists in terms of handling and integration [7,8,9]. In large defects, however, HACaS cements have proven to be insufficiently effective. However, their alternatives also present limitations: although autologous iliac crest cancellous bone is considered the gold standard, it is associated with considerable morbidity at the harvest site. Allogenic grafts reduce this burden but have limitations in terms of availability, immunological safety, and osteogenic potency [10,11]. The healing process of bone defects often remains heterogeneous and prone to revision [11].

In this context, research is increasingly focusing on the biological modulation of the defect environment. A promising approach involves the functionalization of HACaS with antiresorptive (e.g., zoledronic acid, ZA) and osteoanabolic (e.g., bone morphogenic protein 2, BMP-2) agents. While ZA reduces early degradation and preserves primary stability [12], BMP-2 promotes osteogenic regeneration [13]. BMP-2 must be applied locally, whereas ZA can be administered locally or systemically. Research comparing local and systemic outcomes is ongoing, but currently there is no conclusive evidence favoring the use of one over the other. Nonetheless, it is crucial that bioactive additives do not compromise the material properties of the carrier material: setting kinetics, peak load, workability, and dimensional stability are all relevant in clinical settings. Furthermore, the timing of additive incorporation during mixing, through a brief change in the liquid-to-powder ratio, can have a noticeable effect on early strength.

The Vicat/Gillmore tests are considered the standard reference for objectively characterizing setting behavior [14,15]. Standardized compression and flexural tests have been established to characterize the maximum mechanical load capacities of polymerized bone cements. For polymethylmethacrylate-based bone cements (PMMA), ISO 5833 specifies the determination of compressive strength [16]. Against this background, a Vicat/Gillmore-inspired macro-indentation protocol was used in this study, which transfers the penetration logic to force/stress-based endpoints and thus addresses two clinically relevant questions in one setup: at what force does the surface begin to yield continuously, and what maximum force does the test specimen withstand until breakthrough occurs? This study aimed to investigate the influence of ZA and/or BMP-2 on the setting kinetics and peak load of a clinically used HACaS cement. We hypothesized that the timing of the addition affects early setting behavior and, secondly, that ZA and BMP-2 modulate the material properties in different ways without adversely affecting the peak load in a physiological environment.

## 2. Materials and Methods

### 2.1. Mixing of HACaS, Additives, and Timing

HACaS (Cerament^®^; Bonesupport, Lund, Sweden) was enhanced with a BMP-2 solution (BMP-2, Dibotermin alfa (rhBMP-2), InductOs^®^; Medtronic BioPharma B.V., Heerlen, The Netherlands) and/or ZA solution (Zoledro-Denk, DENK PHARMA GmbH & Co. KG, Munich, Germany). Raina et al. demonstrated that a CaS/HA scaffold of comparable size (6 × 4 mm), loaded with 10–15 μg of rhBMP-2 per defect, yields robust bone formation in a rat critical-size femoral defect. The BMP-2 and ZA concentrations in our study were applied in accordance with the protocol described by Raina et al. [17]. In each group, the additives were applied once immediately (0 min) and once after 2 min. HACaS was homogenized at room temperature on a sterile surface in single-use cups with a sterile spatula. Each batch contained 13.3 mg of gentamicin per 1000 mg of HACaS. The total liquid volume followed the manufacturer’s guidance for plain HACaS and was kept constant at 432.4 µL per 1000 mg. When additives were used, the fraction of NaCl was reduced accordingly (Table 1). Time started at first contact between the powder and liquid (*t* = 0 min). In the 0 min groups, additive solutions were added immediately, along with NaCl. In 2 min conditions, HACaS was first mixed for 2 min with the reduced NaCl fraction only (temporarily lower liquid-to-powder ratio; L/P). Subsequently, the withheld additive solution was added at 2 min.

### 2.2. Specimen Preparation

The mixed cement was filled into planar, custom-made, cylindrical molds (4 mm diameter; 6 mm height). The surfaces were leveled to ensure a planar testing interface. Repeated insertion of a needle, as well as shaking and tapping, ensured that the cylinders did not contain any trapped air bubbles. The 90 min and 24 h measurements were performed after setting in the molds at room temperature. For the endpoint hardness tests (“90 min–End”; “24 h–End”), cylinders were removed from the molds and positioned flat on the test bench so that the indenter made orthogonal contact with the top surface.

### 2.3. Twenty-Four-Hour Storage in Physiological Milieu

For the 24 h endpoints, specimens were transferred into fresh human blood (donated voluntarily from the author), representing a physiological environment, and incubated at 37 °C following an initial setting period of 90 min. After 24 h, the samples were removed, gently blotted, and tested.

### 2.4. Indentation/Penetration Testing (Setting and Endpoint Hardness)

#### 2.4.1. Test Concept and Readouts

The setting kinetics were evaluated using a penetration test with a small cylindrical needle indenter mounted on a motorized force tester (MultiTest-dV 2.5 d; Mecmesin Limited, Slinfold, West Sussex, UK). Resistance was recorded as the force required to reach a pre-defined depth. The force readout was provided by a digital force gauge (0–50 N; Mecmesin Limited, Slinfold, West Sussex, UK). Endpoint hardness was defined as the peak force at failure using a larger flat-ended cylindrical indenter with a higher-range force gauge (0–1000 N; Mecmesin Limited, Slinfold, West Sussex, UK). Forces *F* (N) were converted to nominal stress σ (MPa) using the projected area A of the indenter:

$$\sigma \;\left(MPa\right)=\frac{F\;(N)}{A\;({mm}^{2})}$$

#### 2.4.2. Indenter Geometries

Needle indenter: Radius *r* = 0.8 mm; area *A* = *πr^2^* = 2.01 mm^2^.

Cylindrical indenter: Radius *r* = 2.0 mm; area *A* = *πr^2^* = 12.56 mm^2^.

#### 2.4.3. Procedure

Cylindrical cement pellets (Ø 4 mm, height 6 mm) were tested statically at room temperature. For the setting assessment, a small cylindrical needle indenter was advanced into the 6 mm cement bead to a constant penetration depth of 3 mm at a predetermined speed. The maximum force recorded during penetration was converted to MPa.

The endpoint hardness was determined using a larger flat-ended cylindrical punch by increasing the load until failure. The peak force at failure was recorded and converted to nominal stress (MPa).

For the needle-based setting measurements, the force gauge readout was limited to 50 N. Thus, the maximum measurable stress was ≈24.88 MPa (50 N/2.01 mm^2^). For the endpoint hardness (flat punch), a separate device with a higher range was used, so no instrument ceiling affected the measurements.

### 2.5. Statistics

Measurements were taken at pre-defined time points (3, 6, 10, 15, 30, 60, and 90 min; “90–End”; “24 h–End”). Each point was independently analyzed. Each group comprised three replicates (*n* = 3) for the minute-wise panels. The endpoint comprised five replicates (*n* = 5) per group, except for BMP-2 (0 min) at 24 h (*n* = 4). Given the small sample size, all analyses were considered exploratory. For the 3–90 min boxplots, a one-way ANOVA was performed at each time point with each group as an independent factor. Post hoc pairwise comparisons were made using Tukey’s HSD (family wise α = 0.05). Tukey-adjusted *p*-values are displayed as bracket annotations with stars. Endpoint analyses (90 min, 24 h) followed the same scheme (one-way ANOVA across six groups, followed by Tukey’s HSD; stars in the figure). The median time course with IQR ribbons is descriptive and intended for visualization only (no formal inference on trajectories). The model assumptions were checked using residual diagnostics and variance checks. Given the small, largely balanced group sizes, ANOVA is reasonably robust to moderate nonnormality. The results were two-sided with α = 0.05. All analyses were conducted in R 4.5.2 (Windows RGui, Foundation, Wien, Österreich) using base stats and supplementary routines implemented in the project script.

## 3. Results

### 3.1. Additive Composition and Timing Drive Setting Kinetics

**Figure 1:**
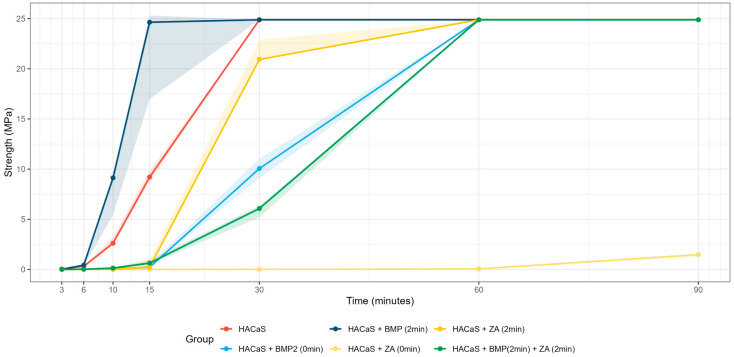
Time course of setting: median indentation values (MPa) over time for HACaS (reference) and additive/timing groups: ZA (zoledronic acid) (0 min), ZA (2 min), BMP-2 (bone morphogenic protein) (0 min), BMP-2 (2 min), BMP-2 (2 min), and +ZA (2 min). Lines = group medians. Shaded area = 95% CI. Two-minute conditions show an earlier rise in strength. ZA (0 min) markedly suppresses early setting (low values up to 90 min). *n* = 3 per group/time point.

**Figure 2:**
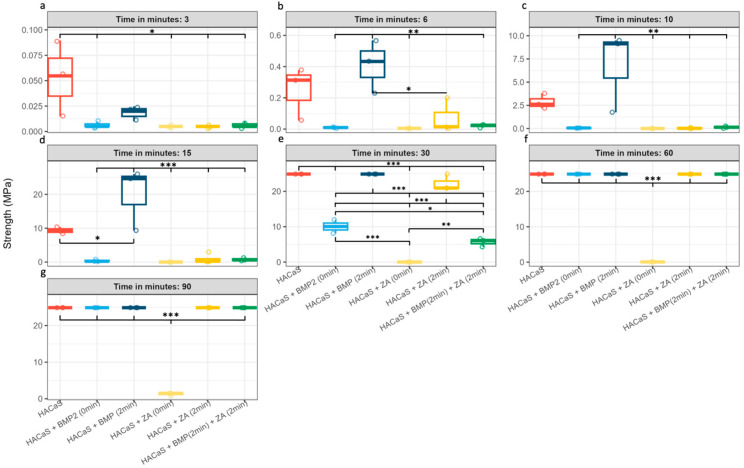
Time-resolved group comparisons: boxplots are grouped for each minute (3–90 min) with Tukey’s HSD post hoc contrasts (ANOVA-based). Subfigures (**a**–**g**) show the maximum hardness at 3 (**a**), 6 (**b**), 10 (**c**), 15 (**d**), 30 (**e**), 60 (**f**) and 90 (**g**) min. The red boxplot represents HACaS (Hydroxyapatite-calcium sulfate), the light and dark blue boxplots represent groups containing HACaS and BMP-2 (bone morphogenic protein 2), the yellow boxplots represent groups containing HACaS and ZA (zoledronic acid) and the green boxplot represents the group containing HACaS, BMP-2 and ZA. The omnibus ANOVA was significant at 3, 6, 10, 15, 30, 60, and 90 min. Numerous adjusted pairwise differences are marked. Star coding: *p* * ≤ 0.05; ** ≤ 0.01; and *** ≤ 0.001. *n* = 3 per group/time point.

The time-resolved medians diverged within the first few minutes of setting (Figure 1). Omnibus ANOVA suggested group differences at all time points (3, 6, 10, 15, 30, 60, and 90 min) although these results should be interpreted cautiously given the small sample size (*n* = 3). The corresponding adjusted post hoc contrasts are detailed in Figure 2 (*n* = 3 per group/time point for the 3–90 min series). The significant group differences should be cautiously interpreted, due to low n number. Later needle-based time points should be interpreted with caution, as several groups approached the upper measurement limit of the 50 N force gauge, which reduces discrimination once the ceiling is reached.

### 3.2. Delayed Addition Enhances Early-Phase Mechanical Properties

All groups prepared using the delayed addition protocol (2 min) showed an earlier increase in hardness than their immediate addition (0 min) counterparts. Notably, the combination of HACaS with BMP-2 added at 2 min appeared to exhibit enhanced early setting performance at *t* = 6 min and *t* = 10 min, achieving significantly higher hardness than all other experimental groups (*p* < 0,01), except for the native HACaS control (Figure 2b,c). At *t* = 15 min, the HACaS + BMP-2 (2 min) group also showed higher values compared to the HACaS control (*p* < 0.05) (Figure 2d). After 30 min, the differences between the two groups leveled out, reaching the highest measured strength among all groups. The HACaS + ZA (2 min) group also demonstrated comparatively high hardness values after 30 min, suggesting improved performance than the HACaS + BMP-2 (0 min), HACaS + BMP-2 (2 min) + ZA (2 min), and HACaS + ZA (0 min) groups (*p* < 0.001) (Figure 2e).

### 3.3. Zoledronic Acid Impedes Early Hardening

**Figure 3:**
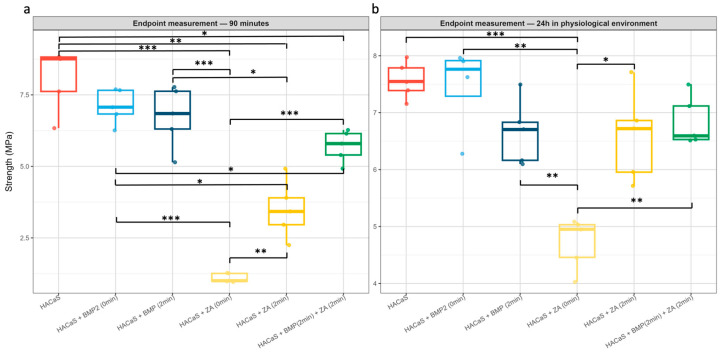
Endpoint hardness: 90 min (**a**) and 24 h in physiological milieu (**b**). Boxplots (median, IQR, and whiskers) with jittered points. Horizontal bars/stars indicate adjusted post hoc comparisons (ANOVA/Welch → Tukey HSD). The red boxplot represents HACaS (Hydroxyapatite-calcium sulfate), the light and dark blue boxplots represent groups containing HACaS and BMP-2 (bone morphogenic protein 2), the yellow boxplots represent groups containing HACaS and ZA (zoledronic acid) and the green boxplot represents the group containing HACaS, BMP-2 and ZA. At 90 min, the differences were pronounced (highest: HACaS and BMP-2 (2 min); lowest: ZA (0 min), ZA (2 min), and combinations in between). At 24 h, the values converged, with ZA (0 min) remaining the lowest. Star coding: *p* * ≤ 0.05; ** ≤ 0.01; and *** ≤ 0.001. *n* = 3 per group/time point. Typical *n* = 5 per group (one endpoint group, *n* = 4).

The immediate addition of ZA was associated with markedly reduced setting. Even at 90 min, the ZA (0 min) group showed negligible crystallization compared with all other groups (Figure 2g). The endpoint measurements confirmed this observation, as the ZA (0 min) group displayed lower hardness values in comparison with all other conditions (*p* < 0.01 compared with HACaS + ZA (2 min) and HACaS + BMP-2 (2 min) + ZA (2 min) and *p* < 0.001 compared with all other groups) (Figure 3).

### 3.4. Combinations with ZA Delay Setting Relative to BMP-2 Alone

The combination containing both BMP-2 and ZA appeared to exhibit a slower rate of strength acquisition than the BMP-2 single additive protocols, regardless of the timing of the addition (0 or 2 min groups). Multiple adjusted pairwise comparisons confirmed the delayed setting behavior in the minute-based analyses (Figure 2). For instance, HACaS + BMP-2 (2 min) + ZA (2 min) only reaches higher strength values after 60 min (Figure 2f).

### 3.5. Endpoint Analysis: Divergence at 90 min; Partial Convergence at 24 h

At the 90 min interval, different group stratifications became apparent: the HACaS control and BMP-2 (2 min) achieved the highest values, whereas ZA (0 min) remained the lowest (Figure 3a). After 24 h of incubation in a physiological milieu, the values largely converged, although the ZA (0 min) group remained significantly inferior (Figure 3b). HACaS + BMP-2 (0 min) and HACaS + BMP-2 (2 min) present similar endpoint hardness at 90 min. After 24 h of incubation in blood, the values tended to diverge slightly with higher hardness values in the HACaS + BMP-2 (0 min) group, without reaching statistical significance.

## 4. Discussion

### 4.1. Liquid-to-Powder Ratio Influences Setting Kinetics

The present exploratory investigation shows that additives and the timing of their addition significantly influence the setting kinetics of HACaS. This can be attributed to two variables: physical changes in the L/P ratio and chemical effects of delayed adjuvant incorporation. Two-minute protocols yielded superior early strength compared with immediate adjuvant addition. The accelerated early setting observed in the 2 min protocols is therefore likely dominated by the transiently reduced L/P ratio, as all 2 min groups consistently exhibited faster setting than their 0 min counterparts, independent of the specific adjuvant (BMP-2 or ZA). This is plausible from a methodological perspective: the initially reduced liquid volume increases the viscosity and cohesion of the paste and promotes early, stable “skin formation” on the surface. The fact that the L/P ratio significantly controls the setting time and early mechanics of HACaS cements is well established in material science: a higher L/P ratio (more liquid) prolongs the setting time and reduces early strength, whereas a low L/P ratio accelerates setting and enhances early strength [18,19]. The fact that BMP-2 (2 min) reached higher values earlier than pure HACaS can be primarily attributed to the specific addition protocol, characterized by an initially reduced L/P ratio. However, this contribution cannot be unequivocally isolated, and in the case of the delayed addition of ZA, a later onset of ZA-related inhibitory effects after 2 min may additionally modulate the setting behavior. Future experiments that independently vary the L/P ratio and timing of the addition are required to disentangle these effects.

In clinical practice, it is essential for the cement to quickly achieve enough cohesion and surface stability. It must endure irrigation, exposure to blood, shaping of defects, and wound closure without being washed away or collapsing. Therefore, differences in the first 30–90 min may be relevant for the assessment of early material behavior during cement maturation, although the present in vitro assay does not directly model intraoperative handling or implant stability.

### 4.2. Citrate-Containing Solutions Inhibit Early Setting

**Figure 4:**
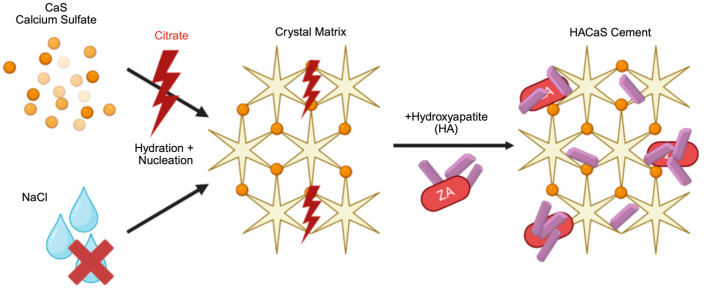
Crystallization process of HACaS cement in presence of citrate. Citrate binds Ca^2+^, reducing calcium activity and inhibiting nucleation and crystal maturation. Zoledronic acid (ZA) binds hydroxyapatite, disrupting crystal growth.

The delayed setting observed with ZA, particularly with the immediate addition at 0 min, is consistent with two processes. The first factor is chelation: Citrate (a component of common ZA infusion solutions, including Zoledro-Denk^®^) binds free Ca^2+^, reducing the available calcium activity, which is analogous to the mechanism underlying transfusion-associated hypocalcemia [20]. When applied to HACaS cements, a citrate-rich environment can inhibit or delay the nucleation and crystal maturation of calcium phosphate/sulfate phases [21]. The second factor is surface complexation: Bisphosphonates bind avidly to hydroxyapatite surfaces. This direct surface complexation disrupts crystal growth, influences surface properties, and impedes early strength development, particularly when intervening during the early mineral formation phases [22,23]. Figure 4 summarizes these two mechanisms. The practical consequence of these chemical interactions is that the timing of the addition and vehicle composition are critical variables for ZA when used in combination with HACaS cements. Importantly, these findings do not argue against the biological concept of antiresorptive support per se but rather against the early local incorporation of citrate-containing ZA solutions. A potential alternative for future preclinical or translational evaluation might be the administration of systemic ZA, which would avoid direct interference with cement mineralization while still providing antiresorptive exposure. Such an approach may be of interest in settings where local BMP-2 delivery is used to stimulate osteogenesis and ZA is considered to counteract early resorption. Further investigation of these combinations, including in vivo models, is required.

In the ZA + BMP-2 combination, the early increase was also flatter than that with BMP-2 alone. These observations are consistent with the overlapping inhibitory effect of ZA on mineral formation. Notably, the ZA + BMP-2 combination approached the performance of the non-ZA groups only after approximately 60 min (Figure 2f). Within the present assay, the ZA + BMP-2 combination showed a prolonged delay in early hardening relative to the non-ZA groups. For improved practical application, ZA should not be added directly to fresh preparations at 0 min or in a citrate-containing vehicle. Delayed addition and/or vehicle adjustment appeared to be more favorable.

### 4.3. Effects of 24 h of Incubation in Blood and Practical Considerations for Potential Translation into Clinical Practice

At 90 min, clear group differences were evident. Following 24 h of incubation in blood, the setting values largely converged; however, those of the ZA (0 min) group remained significantly inferior. The physiological environment (37 °C, proteins/ions) supported further crystallization. However, it did not mitigate initial mixing or substance-specific effects; mixture- and substance-related effects remain detectable. Since early mechanical properties are critical for intraoperative handling and primary stability, the near-complete inhibition of setting observed with immediate ZA addition (0 min) is of particular concern. Consequently, future protocols combining HACaS and ZA must avoid early-phase incorporation to prevent compromising clinical consolidation efficacy.

An exception to the mixture-related effects apparent after 24 h of incubation in blood was the comparison between HACaS + BMP-2 (0 min) and HACaS + BMP-2 (2 min). After 24 h of incubation, the group with immediate BMP-2 addition tended to show slightly increased hardening relative to its counterpart, suggesting potentially better performance under physiological conditions (Figure 3b). This could be relevant for intraoperative use. However, when considering the minute measurements, the immediate-addition group only reached adequate setting values after 60 min, which substantially limits its practicality for implantation.

From a translational perspective, combining a clinically used HACaS cement with BMP-2 and/or bisphosphonates may represent an off-label approach depending on the indication, dose, and delivery route. Therefore, a systematic preclinical assessment of handling, setting kinetics, and early mechanical integrity is essential to mitigate patient risk. The present results provide exploratory data to rationally design pilot preclinical applications and define safe preparation protocols (timing, vehicle, and early handling constraints).

### 4.4. Relation to Standard Methods and Limitations

This macro-indentation protocol is Vicat/Gillmore-inspired and intended as a complementary approach to established reference methods. Therefore, the results should not be interpreted as a substitute for standardized testing or mechanical strength testing. The classical Vicat (ISO 9597) [14] and Gillmore (ASTM C266) [15] tests determine the time-to-set via standardized needle penetration in cement pastes. In contrast, our protocol preserves the penetration logic but reports force/stress-based endpoints with defined indenter geometries (needle for setting; flat cylindrical indenter for endpoint hardness), converting forces to nominal stress (MPa). It is not intended to replace the standard procedures; rather, it provides comparative force-based readouts relevant to early material behavior within the present setup. A schematic comparison of the present study-specific indentation setup with classical Vicat and Gillmore setting tests is provided in Supplementary Figure S1.

From a mechanical perspective, these experiments were performed under static conditions and thus do not replicate the complex, cyclic, and multiaxial loading environment in vivo. Therefore, the present data cannot predict long-term fatigue behavior, failure under physiological load, or the interaction with surrounding bone during functional loading. The 24 h measurement in blood approximates an early postoperative scenario but remains an in vitro model. These comparisons aid clinical classification but do not replace ISO-compatible compression and bending tests, which were absent in this study but are important for simulating the stresses that occur during physiological movements and for establishing further conclusions regarding the practicability of bone cements.

It is important to note that indentation measurements are influenced by indenter geometry, so nominal stress values cannot be regarded as geometry-independent material constants. Therefore, in this study, we primarily compare setting behavior within each indenter type; a direct comparison between absolute values obtained with the needle (minute panels) and flat cylindrical indenters (endpoint) was not appropriate. There are some limitations to this study, such as its small test groups, particularly in the hardening behavior investigations. Therefore, the results should be interpreted as exploratory. Furthermore, the 50 N display limit for the needle measurement was a limiting factor for this study, as some groups reached this limit after only 30 min.

A comparison is helpful to classify the magnitude of the measured maximum force. The upper needle limit of 50 N (≈24.9 MPa) corresponds to approximately 12–19% of the typical compressive strength of the cortical bone (130–200 MPa) and approximately 35% of the minimum compressive strength for PMMA bone cements according to ISO 5833 (≥70 MPa) [16,24,25]. The trabecular bone, with an average compressive strength of 0.1–30 MPa, is thus partially below the maximum value measured in the setting test [24]. These comparisons aid clinical classification but do not replace ISO-compatible compression/bending tests, which were absent in this study. These are important for simulating the stresses that occur during physiological movements and allow for further conclusions to be established regarding the practicability of bone cements.

Regarding the adjuvants, this study specifically examined the effects of BMP-2 and ZA on setting kinetics and early mechanical properties without re-evaluating release kinetics or biocompatibility in the same experimental series. Existing in vitro and in vivo data on comparable CaS/HA–BMP-2–ZA combinations support their general feasibility, and we intend for our findings to be a mechanical and handling-oriented complement to these studies [26]. The experimental concentrations of BMP-2 and ZA were chosen in an exploratory manner based on prior preclinical work, and no concentration-gradient groups were included [17]. Consequently, defining an optimal therapeutic window lies beyond the scope of this study and should instead be addressed in future dose–response investigations.

## 5. Conclusions

In conclusion, additive timing and vehicle composition appear to be important determinants of the early mechanical behavior of augmented HACaS cements. A delayed addition protocol (2 min), which transiently reduced the liquid-to-powder ratio, was associated with improved early strength and may prove the most favorable practical profile for local BMP-2 supplementation. In contrast, early local ZA addition, particularly via citrate-containing infusion vehicles, was associated with impaired setting and may be less suitable. If antiresorptive support is desired, systemic ZA administration or delayed/local citrate-free strategies may represent more compatible approaches for intraoperative handling. These in vitro findings provide an initial experimental basis for future in vivo studies and may help the design of subsequent translational investigations.

## Figures and Tables

**Table 1:** Composition per 1 g of *HACaS* (total liquid = 432.4 µL; ZA = zoledronic acid; BMP-2 = bone morphogenic protein 2).

Group	NaCl [µL]	BMP-2 Solution [µL]	ZA Solution [µL]	BMP-2 [µg]	ZA [µg]
HACaS	432.4	0	0	0	0
HACaS + BMP-2	332.4	100	0	150	0
HACaS + ZA	307.4	0	125	0	100
HACaS + BMP-2 + ZA	207.4	100	125	150	100

## Data Availability

The raw data supporting the conclusions of this article will be made available by the authors on request.

## Supplementary Materials

The following supporting information can be downloaded at: https://www.mdpi.com/article/10.3390/ma19091873/s1, Figure S1: Conceptual comparison of the study-specific indentation setup with classical Vicat and Gillmore setting tests. (A) Study-specific comparative indentation setup used in the present work. Small cylindrical pellet specimens were tested in a force-based setup using a defined indenter geometry. (B) Standardized Vicat setting-time test, which determines setting based on the penetration of a defined needle into a standardized bulk cement paste specimen until a predefined endpoint is reached. In ASTM C191 [27], the paste is prepared from 650 g cement, tested in a 40 mm high Vicat mold, and the Vicat initial setting endpoint is defined by 25 mm penetration, whereas final setting is defined as the first measurement that does not leave a complete circular impression. (C) Standardized Gillmore setting-time test, which determines initial and final setting using weighted needles and predefined penetration endpoints in standardized bulk cement paste specimens. In ASTM C266, the paste is likewise prepared from 650 g cement, the initial Gillmore needle is 113.4 g with a 2.12 mm tip, and the final Gillmore needle is 453.6 g with a 1.06 mm tip. Initial and final setting are each defined as the first measurement at which the respective needle does not leave a complete circular impression. These methods differ in specimen size, endpoint definition, and intended purpose.

## Abbreviations

The following abbreviations are used in this manuscript:

A	Area
BMP-2	Bone morphogenic protein 2
F	Force
HACaS	Hydroxyapatite–calcium sulfate
L/P	Liquid-to-powder
min	Minutes
mm	Millimeters
MPa	Mega Pascal
N	Newton
PMMA	Polymethyl methacrylic
r	Radius
ZA	Zoledronic acid
