# Supplementary material for: Adjuvants Alter the Setting Behavior of a Ceramic Bone Graft Substitute: Implications for the Laboratory and Operating Room
Source: Materials (Basel). 2026 May 1;19(9):1873. doi: 10.3390/ma19091873 (PMC13165178; doi:10.3390/ma19091873)
Supplement: Supplementary file 1 [file materials-19-01873-s001.zip › materials-4254043-supplementary.pdf]

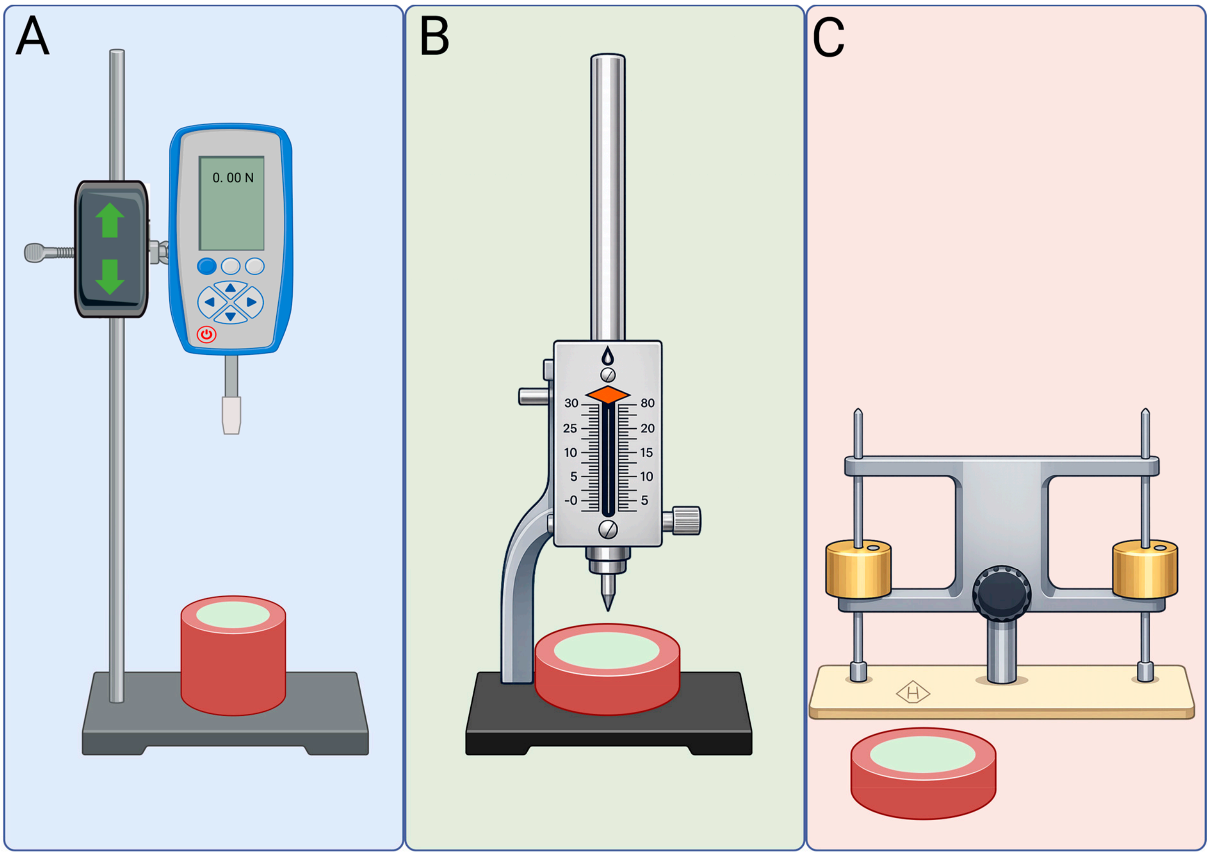

Supplementary Figure S1: Conceptual comparison of the study-specific indentation setup with classical Vicat and Gillmore setting tests. (A) Study-specific comparative indentation setup used in the present work. Small cylindrical pellet specimens were tested in a force-based setup using a defined indenter geometry.
(B) Standardized Vicat setting-time test, which determines setting based on the penetration of a defined needle into a standardized bulk cement paste specimen until a predefined endpoint is reached. In ASTM C191, the paste is prepared from 650 g cement, tested in a 40 mm high Vicat mold, and the Vicat initial setting endpoint is defined by 25 mm penetration, whereas final setting is defined as the first measurement that does not leave a complete circular impression.
(C) Standardized Gillmore setting-time test, which determines initial and final setting using weighted needles and predefined penetration endpoints in standardized bulk cement paste specimens. In ASTM C266, the paste is likewise prepared from 650 g cement, the initial Gillmore needle is 113.4 g with a 2.12 mm tip, and the final Gillmore needle is 453.6 g with a 1.06 mm tip. Initial and final setting are each defined as the first measurement at which the respective needle does not leave a complete circular impression.
These methods differ in specimen size, endpoint definition, and intended purpose.
